# NAD-Linked Metabolism and Intervention in Short Telomere Syndromes and Murine Models of Telomere Dysfunction

**DOI:** 10.3389/fragi.2021.785171

**Published:** 2021-10-27

**Authors:** Amanda J Stock, Yie Liu

**Affiliations:** Laboratory of Genomics and Genetics, Biomedical Research Center, National Institute on Aging/National Institutes of Health, Baltimore, MD, United States

**Keywords:** short telomere syndromes, telomerase null mice, NAD metabolism, DNA damage response, CD38 NADase, PARPs, SIRT1, mitochondrial dysfunction

## Abstract

Telomeres are specialized nucleoprotein structures that form protective caps at the ends of chromosomes. Short telomeres are a hallmark of aging and a principal defining feature of short telomere syndromes, including dyskeratosis congenita (DC). Emerging evidence suggests a crucial role for critically short telomere-induced DNA damage signaling and mitochondrial dysfunction in cellular dysfunction in DC. A prominent factor linking nuclear DNA damage and mitochondrial homeostasis is the nicotinamide adenine dinucleotide (NAD) metabolite. Recent studies have demonstrated that patients with DC and murine models with critically short telomeres exhibit lower NAD levels, and an imbalance in the NAD metabolome, including elevated CD38 NADase and reduced poly (ADP-ribose) polymerase and SIRT1 activities. CD38 inhibition and/or supplementation with NAD precursors reequilibrate imbalanced NAD metabolism and alleviate mitochondrial impairment, telomere DNA damage, telomere dysfunction-induced DNA damage signaling, and cellular growth retardation in primary fibroblasts derived from DC patients. Boosting NAD levels also ameliorate chemical-induced liver fibrosis in murine models of telomere dysfunction. These findings underscore the relevance of NAD dysregulation to telomeropathies and demonstrate how NAD interventions may prove to be effective in combating cellular and organismal defects that occur in short telomere syndromes.

## Introduction

**FIGURE 1:**
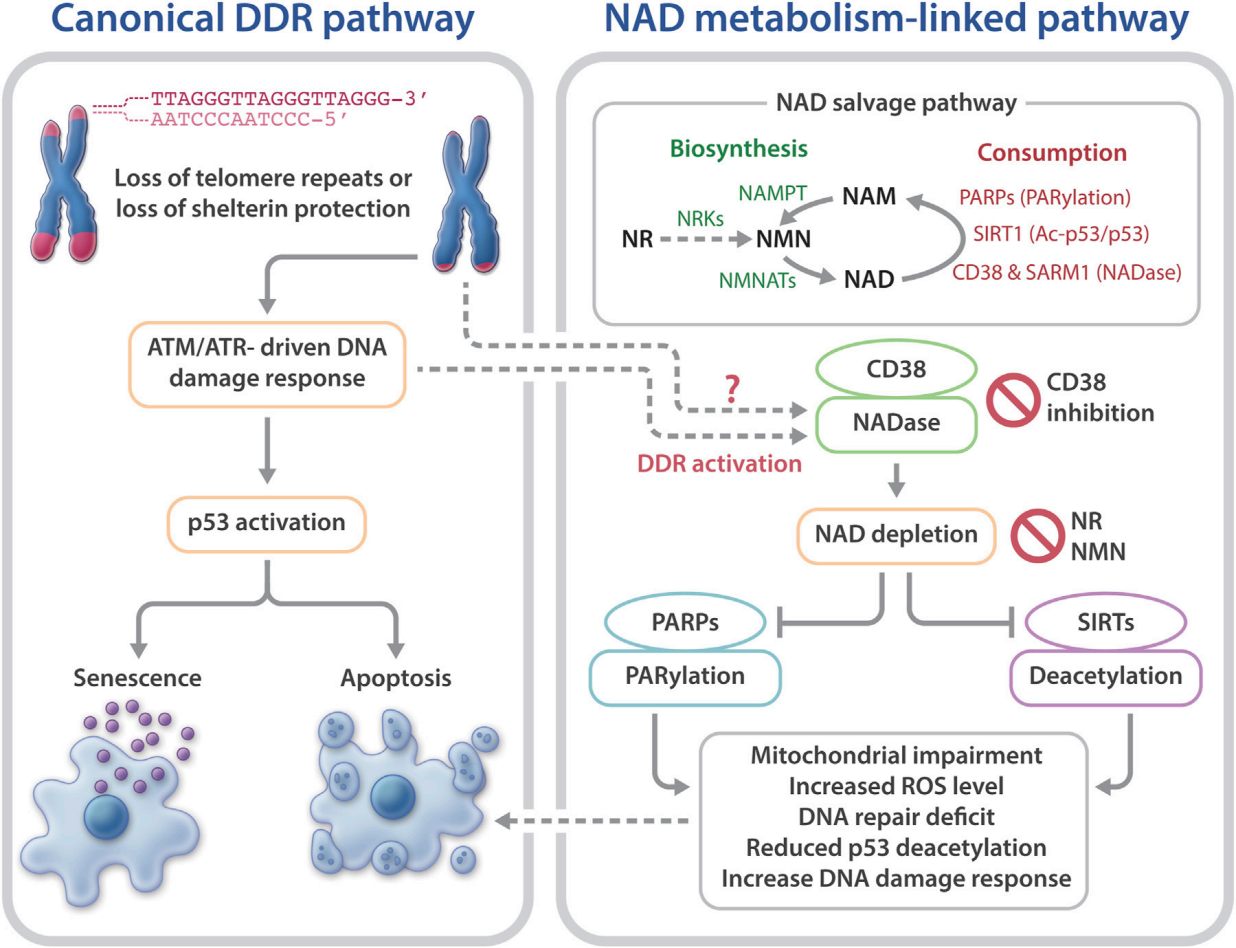
Telomere dysfunction-induced DNA damage response and NAD metabolism in telomeropathies. Telomere dysfunction via loss of telomere repeats or loss of protection by telomeric shelterin proteins elicits DDR, driving cellular senescence or apoptosis. Short telomeres and DDR also evoke NAD metabolome dysregulation, via CD38 hyperactivation, which excessively consumes NAD and reduces the available NAD to PARPs and SIRTs. As a result, the functions of PARylation and SIRT1 activities in telomere and mitochondrial maintenance are limited. Consequently, mitochondrial impairment-induced ROS and defective PARP-related DNA repair may accelerate telomere damage and aggravate cellular senescence or apoptosis. Top right: an overview of the NAD salvage pathway. The NAD consuming enzymes, PARPs, SIRTs, and NADases consume NAD and generate NAM. The NAD biosynthesizing enzyme nicotinamide/nicotinic acid mononucleotide adenylyltransferase (NMNAT) recycles NAM into NMN, followed by conversion of NMN to NAD by nicotinamide mononucleotide (NAMPT). The NAD precursor, NR is converted to NMN by NRKs nicotinamide riboside kinases.

Telomeres are chromosome termini structures consisting of tandem DNA nucleotide repeats and a six-protein complex (shelterin complex) composed of TRF2, TRF1, POT1, TIN2, TPP1, and RAP1 (De Lange, 2018). Telomeres cap chromosome ends from eliciting the DNA damage response (DDR) and play a central role in monitoring cell divisions and ensuring genome integrity. Loss of telomere repeats or loss of protection by the shelterin complex can evoke an ATM- or ATR-dependent DDR, resulting in telomere dysfunction-induced foci containing γ-H2AX and other DDR proteins (d’adda di Fagagna et al., 2003; Takai et al., 2003). This telomere-dysfunction induced DDR is a driver of cellular senescence, cell death, and genome instability (d’adda di Fagagna et al., 2004) (Figure 1).

NAD (or NAD^+^) and its reduced form NADH are fundamental coenzymes in redox reactions required for energy metabolism. Depletion of NAD has emerged as a critical feature of aging (Canto et al., 2015; Verdin, 2015; Chini et al., 2017; Fang et al., 2017). The bioavailability of NAD is diminished in aged and DNA repair-deficient animal models with premature aging phenotypes, and treatment of these models with NAD precursors has demonstrated improvements in mitochondrial integrity, genome stability, and health span (Fang et al., 2014; Scheibye-Knudsen et al., 2014; Mills et al., 2016; Das et al., 2018; Mitchell et al., 2018; Fang et al., 2019; Igarashi et al., 2019; Tarantini et al., 2019; Okur et al., 2020).

Recent studies have begun to shed light on the role of critically short telomeres in NAD dysregulation (Sun et al., 2020). Additionally, these studies have begun to show that NAD-boosting supplements provide benefits for DC and murine models with critically short telomeres (Amano et al., 2019; Sun et al., 2020). In this review, we discuss the molecular basis linking short telomeres to imbalanced NAD metabolism as well as the evidence supporting that NAD intervention could be utilized to combat the pathological consequences of telomere shortening/dysfunction (or telomeropathies).

### Telomere Loss in Cellular Senescence and Short Telomere Syndromes

As one of the hallmarks of aging (Lopez-Otin et al., 2013), telomere attrition is associated with biological/chronological aging (Blackburn et al., 2015; Chakravarti et al., 2021). This association underscores the importance of telomere maintenance in aging and age-related disease. Short telomeres are a key feature of short telomere syndromes including DC, aplastic anemia, and idiopathic pulmonary fibrosis (Armanios, 2013; Sarek et al., 2015; Bertuch, 2016; Savage, 2018). Patients with DC have very short telomeres at a young age due to germline mutations of key telomere maintenance genes, and have a high risk for bone marrow failure, cancer, pulmonary fibrosis, liver disease, and numerous other conditions (Dokal, 2011; Savage, 2018; Bhala et al., 2019). Emerging evidence suggests a crucial role for critically short telomere-induced DDR in cellular dysfunction in DC (Armanios, 2013; Bertuch, 2016; Savage, 2018). However, other pathways or cellular defects cannot yet be ruled out as contributing factors in the pathophysiology of DC. For example, DC patients and late generation telomerase null mice display mitochondrial impairment (Sahin et al., 2011; Westin et al., 2011), which may further increase telomere DNA damage, thereby accelerating telomere dysfunction. A prominent factor linking nuclear DNA damage to mitochondrial homeostasis is the nicotinamide adenine dinucleotide (NAD) metabolite. NAD is utilized by poly (ADP-ribose) polymerases (PARPs) and sirtuins (SIRTs), and the NAD-dependent activities of PARPs and SIRTs participate in signaling networks that are involved in mitochondrial health, genome maintenance, and longevity (Canto et al., 2015; Verdin, 2015; Fang et al., 2017). Detailed characterization of NAD metabolism as well as the underlying molecular mechanisms linking the NAD metabolite to mitochondrial impairment in DC and mouse models of telomere dysfunction remain elusive.

### NAD Metabolism in Aging and Diseases

NAD is an essential coenzyme for various metabolic pathways, including glycolysis, fatty acid oxidation, and the tricarboxylic acid cycle. Depletion of NAD occurs with aging and in various age-related pathologies, including liver disease, cardiomyopathy, sarcopenia, and neurodegenerative diseases (Canto et al., 2015; Verdin, 2015; Chini et al., 2017; Fang et al., 2017; Katsyuba et al., 2020). The mechanisms leading to the loss of NAD in many of these pathologies are not well known. Efficient synthesis and consumption of NAD requires various enzymes, and disruption of these enzymes could distort the biological pathways that utilize NAD.

NAD is synthesized via the *de novo* biosynthesis, Preiss-Handler, and salvage pathways (Canto et al., 2015; Verdin, 2015; Fang et al., 2017). Intracellular NAD levels are greatly impacted by the NAD salvage pathway that consists of several NAD consumption and biosynthesis enzymes (Canto et al., 2015; Verdin, 2015; Chini et al., 2017; Fang et al., 2017) (Figure 1). The NAD-biosynthesizing enzymes recycles the NAD precursor nicotinamide (NAM) into nicotinamide mononucleotide (NMN), which is then adenylated to NAD. NMN can also be generated from the NAD precursor, nicotinamide riboside (NR) by nicotinamide riboside kinases (NRKs) (Bieganowski and Brenner, 2004). Nicotinamide phosphoribosyltransferase (NAMPT) is believed to be the rate limiting NAD biosynthesizing enzyme, and its expression declines with aging, which leads to a decreased intracellular NAD concentration (Revollo et al., 2004; Wang et al., 2006; Imai and Yoshino, 2013; Ma et al., 2017). In contrast to NAM, NMN and NR do not require NAMPT to generate NAD (Figure 1), which may make them more attractive therapeutic candidates for age-related diseases and telomeropathies.

NAD is consumed mainly by three groups of NAD-consuming enzymes: PARPs, SIRTs, and NADase/cyclic ADP-ribose synthases (Canto et al., 2015; Verdin, 2015; Chini et al., 2017;Fang et al., 2017). SIRTs are a family of NAD-dependent deacylases. Seven different SIRTs are expressed in mammals, with SIRT1 being the most characterized consumer of NAD (Morris, 2013; Canto et al., 2015). PARPs are a group of proteins that catalyze the transfer of the first ADP-ribose unit from NAD to target molecules, and PARP1 accounts for ∼90% PARP activity (D’amours et al., 2001; Kim et al., 2005). The third group of NAD-consuming enzymes are NADase/cyclic ADP-ribose synthases, which include CD38 and sterile alpha and Toll/interleukin receptor motif-containing protein 1 (SARM1) (Gerdts et al., 2015; Gerdts et al., 2016; Chini et al., 2017; Katsyuba et al., 2020). The NADases hydrolyze NAD to produce NAM and ADP-ribose or a precursor of the second messenger molecule, cyclic ADP-ribose. The NAM by-product generated from NAD consumption reactions is recycled back to NAD by the biosynthesizing enzymes (Figure 1).

NAD consuming enzymatic reactions are vital for signaling networks that contribute to cell fate, including mitochondrial and genome maintenance (Canto et al., 2015; Verdin, 2015; Chini et al., 2017; Fang et al., 2017). The major PARP family protein, PARP1, plays a critical role in genome maintenance and is involved in DNA repair (Kim et al., 2005; Gibson and Kraus, 2012; Eisemann and Pascal, 2019). NAD consumption by PARP1 is mostly increased due to poly (ADP) ribosylation (PARylation) during genotoxic stress (Kruger et al., 2020). Persistent activation of PARP1 has been shown to cause a significant decrease in cellular NAD in DNA repair-deficient rodent and human cells (Fang et al., 2014; Fang et al., 2016). The major sirtuin deacetylase, Sirtuin 1 (SIRT1) participates in the deacetylation and activation of several proteins implicated in the regulation of DDR and mitochondrial homeostasis, including p53 and the peroxisome proliferator-activated receptor γ coactivator-1α (PGC-1α) (Fernandez-Marcos and Auwerx, 2011; Morris, 2013; Tang, 2016). Increasing NAD levels enhances SIRT1 activity, leading to increased PGC-1α expression and mitochondrial biogenesis (Barbosa et al., 2007; Bai et al., 2011; Canto et al., 2012; Gong et al., 2013; Yang et al., 2014; Yang and Sauve, 2016). CD38 is the predominant NADase in mammalian cells and establishes intracellular NAD levels (Chini, 2009; Chini et al., 2017). CD38 expression and activity increases with aging and may contribute to age-related NAD decline (Camacho-Pereira et al., 2016). However, the mechanisms underlying this age-dependent increase in CD38 expression are unclear. CD38 hyperactivity contributes to a decline in PARylation and SIRT deacylase activities, resulting in mitochondrial impairment and genome instability in aged mice (Camacho-Pereira et al., 2016; Chini et al., 2017). Conversely, depletion or inhibition of CD38 leads to significantly increased NAD concentrations and ameliorates age-related metabolic and immune dysfunction in mice (Chini, 2009; Camacho-Pereira et al., 2016; Chini et al., 2018; Hogan et al., 2019; Chini et al., 2020). Besides CD38, another NADase, SARM1, also participates in regulating intracellular NAD levels (Gerdts et al., 2015; Gerdts et al., 2016; Summers et al., 2016; Essuman et al., 2017). Notably, NAD-consuming enzymes compete for bioavailable NAD (Camacho-Pereira et al., 2016). Thus, hyperactivity of one NAD-consuming enzyme may limit the activities of the others, consequently skewing their cellular functions.

### Nicotinamide Adenine Dinucleotide Metabolism and Intervention in Short Telomere Syndromes and Murine Models of Telomere Dysfunction

**FIGURE 2:**
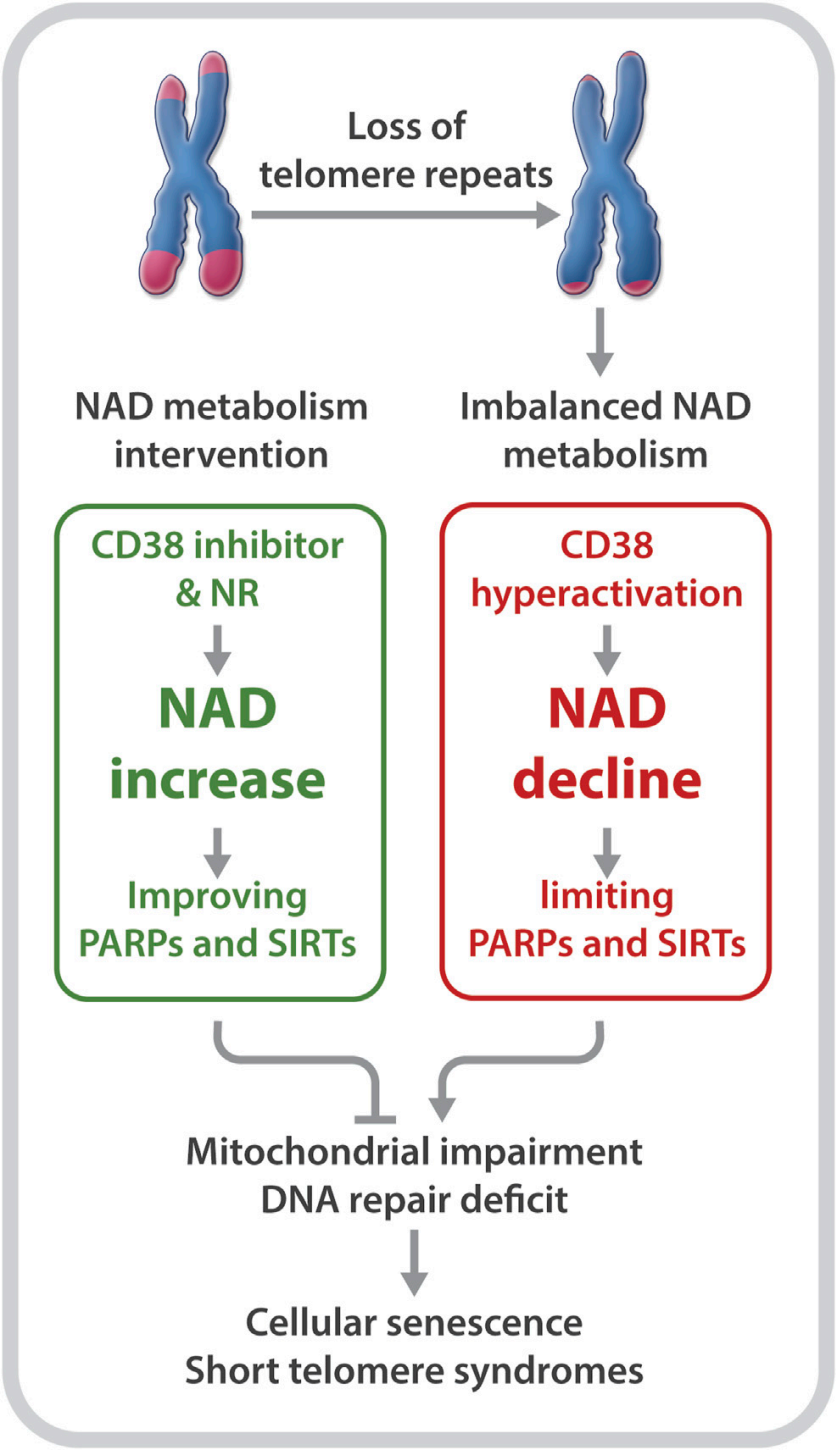
Short telomeres evoke an imbalance in NAD metabolism in primary cells derived from patients with DC. NR supplementation and CD38 inhibition improve NAD homeostasis and the biological pathways regulated by PARPs and SIRTs, which diminish telomere DNA damage and mitochondrial impairment, and delay replicative senescence.

Declines in telomere length and in NAD levels have emerged as key features of aging (Mouchiroud et al., 2013; Verdin, 2015; Zhu et al., 2015; Camacho-Pereira et al., 2016; Fang et al., 2017; Katsyuba et al., 2020). However, the molecular basis linking these hallmarks of aging is unclear. Recently, our group utilized primary fibroblasts derived from DC patients as well as late generation telomerase reverse transcriptase knockout (*Tert* ^*−/−*^) mice with critically short telomeres to investigate the relationship between critically short telomeres/telomere dysfunction and the NAD metabolome (Sun et al., 2020). Our studies revealed several insights: 1) DC fibroblasts and late generation *Tert* ^*−/−*^ mice are defective in NAD metabolism. This is evident based on lower NAD levels and elevated CD38, but reduced PARP1 and SIRT1 expression and activities; 2) DC fibroblasts are defective in the NAD-consuming enzyme-related signaling networks, including the SIRT1-mitochondria and the PARP-telomere DNA repair axis (Figure 1); and 3) NAD mechanism-based intervention strategies restore NAD levels and/or NAD-consuming PARP and SIRT activities, alleviate mitochondrial impairment, and delay the onset of replicative senescence in DC fibroblasts (Figure 2).

In line with these findings, a recent report by Amano et al. demonstrated decreased SIRT expression and activity in the livers of late generation *Tert* ^*−/−*^ mice. In addition, supplementation with the NAD precursor, NMN, led to increased expression of SIRT1 and ameliorated carbon tetrachloride- and thioacetamide (TAA)-induced liver fibrosis in late generation *Tert* knockout mice (Amano et al., 2019). These findings signify a potential benefit of NAD metabolism-based interventions in alleviating the liver pathology that occurs in patients with short telomere syndromes.

Boosting the NAD metabolite may not only alleviate the NAD-related biological pathways that are disrupted by telomere shortening/dysfunction, but may also reduce telomere damage to prevent a further imbalance of the NAD-metabolome. Tarragó et al. observed increased NAD levels, accompanied by fewer telomere dysfunction-induced foci in hepatocytes and myofibers of aged mice given the CD38 inhibitor, 78c (Tarrago et al., 2018). Our studies revealed a decreased number of telomeric oxidative lesions and telomere dysfunction-induced foci in DC fibroblasts upon treatment with NR (Sun et al., 2020). The decreased telomere DNA damage may be dependent on improved functions of SIRTs and PARPs, since NR supplementation and CD38 inhibition could restore their activities (Sun et al., 2020). In addition, NR treatment significantly dampened the DNA damage response, including p53 acetylation as well as p16 and p21 expression in DC fibroblasts (Sun et al., 2020). Therefore, rescuing NAD levels and/or diverting its consumption from CD38 to PARPs and SIRTs could improve telomere integrity, which in turn, could diminish telomere shortening/dysfunction-induced NAD dysregulation.

Collectively, these studies support the notion that telomere shortening/dysfunction in both humans and mice interferes with the NAD metabolism, i.e., a decline in telomere repeats leads to CD38 NADase hyperactivation. Subsequently, CD38 hyperactivity drives the loss of NAD homeostasis, thereby limiting NAD bioavailability for PARP and SIRT enzymatic activities. Decreased PARP and SIRT activities would disrupt their related biological pathways, thereby aggravating telomere/genome damage and mitochondrial abnormalities, which ultimately contributes to cellular senescence and telomeropathy (Figure 1).

### Future Aspects

Recent studies have resolved several key questions, including 1) if telomere shortening disrupts NAD metabolism; 2) if defective NAD metabolism contributes to telomere disease features; and 3) if supplementation of NAD precursors rescues the telomere disease features. Aspects to be further explored include: 1) tissue-specific differences in the benefits provided by NAD re-equilibration in the context of telomere shortening/dysfunction; 2) the molecular pathways linking telomere shortening/dysfunction and dysregulated NAD consuming enzymes, e.g., CD38 hyperactivation; 3) Is CD38 hyperactivation responsible for telomere shortening/dysfunction-mediated pathophysiology in other short telomere syndromes and animal models? and 4) does re-equilibration of imbalanced NAD metabolome ameliorate pathophysiology in patients with short telomere syndromes*?*

It is conceivable that highly proliferative organs, such as bone marrow, skin, and reproductive systems that are more susceptible to critically short telomere-induced DDR would benefit from NAD interventions. Thus, it would be of great interest to explore the tissue-specific effects of NAD interventions on health-span and telomere integrity and function over time. Previous reports have demonstrated beneficial effects of NAD supplementation on hematopoiesis (Vannini et al., 2019). Since bone marrow failure is the leading cause of mortality in patients with DC, it would be valuable to investigate the potential therapeutic value of NAD intervention for critically short telomere-elicited bone marrow failure in mouse models with telomere dysfunction.

Although the molecular basis leading to CD38 overexpression in aging is not fully understood, ATM inhibition leads to a partial decline in CD38 levels in DC fibroblasts (Sun et al., 2020). Thus, a DDR-dependent mechanism may contribute to CD38 hyperactivation in DC. In addition, telomere dysfunction-induced p53 activation represses the expression of SIRTs, while supplementation with the NAD precursor, NMN improves SIRT1 deacetylation activity (Amano et al., 2019). Thus, it is likely that both telomere dysfunction-induced CD38 and p53 activation may drive the loss of NAD homeostasis.

NAD mechanism-based intervention strategies via supplementation with the NAD precursors, NR and NMN, restore NAD levels and homeostasis, delay cellular senescence, and improve health-span in DC fibroblasts and in telomerase null mice (Amano et al., 2019; Sun et al., 2020). Emerging evidence has also identified CD38 as a potential target in combating cancer and age-related diseases (Chini, 2009; Chini et al., 2018; Manna et al., 2019). Although CD38 knockdown leads to only a moderate increase in intracellular NAD levels in DC fibroblasts, it boosts the crucial NAD consumption activities of PARPs and SIRT1 (Sun et al., 2020). Similarly, the CD38 inhibitor, 78c, moderately increases intracellular NAD levels in DC fibroblasts (Sun et al., 2020). Since PARylation and SIRT1 deacetylation activities are particularly low in DC fibroblasts, NAD is likely consumed by restored activities of PARPs and SIRT1 upon CD38 depletion. When combined with a low dose of NMN, CD38 depletion or inhibition resultes in significantly elevated NAD levels (Sun et al., 2020). Thus, a combination of CD38 inhibition and NAD supplementation may present as an effective intervention for human telomere-driven diseases.
